# Subtalar Arthroereisis as a Reliable Option for Flexible Flatfoot in Children – A Case Series

**DOI:** 10.5704/MOJ.2511.006

**Published:** 2025-11

**Authors:** F Miraj, IWAM Karda, EA Noor

**Affiliations:** Department of Orthopaedics and Traumatology, Universitas Indonesia, Jakarta, Indonesia

**Keywords:** flexible flatfoot, subtalar arthroereisis, radiological, functional outcome, case series

## Abstract

**Introduction:**

Conservative treatment in flexible flat foot (FFF) is preferred in most cases; only in symptomatic and conservative-refractory cases is surgical treatment proposed. Subtalar arthroereisis (STA) is a well-established technique, but there remains a paucity of information surrounding the effectiveness and outcome. The aim of this study was to evaluate radiological and functional outcome of STA in FFF.

**Material and Methods:**

This study is a case series of 15ft with symptomatic FFF treated by STA from a period of 2018-2024. Mean age at the time of surgery was 10.1±0.83 years. There was a significant increase in both American Orthopaedic Foot and Ankle Society score (AOFAS) and The Oxford Ankle Foot Questionnaire for Children (OxAFQ-C) subjective scoring with p-value of <0.001. Degree of valgus correction was statistically significant (p=0.001). There were significant differences between pre-and post-operative lateral radiographic measurements with p-value of <0.005. One patient with the lowest radiological angle of correction and increased femoral anteversion had loosened implant in two years follow-up.

**Results:**

Despite results in earlier studies, recent evidence, including randomised control trials, supports the reliability of STA in managing FFF, advocating its use over traditional osteotomies due to fewer complications and quicker rehabilitation periods. In our study, all subjects had significant improvement in lateral radiographic measurements and subjective scoring evaluation during follow-up.

**Conclusion:**

STA is an option for surgical treatment in symptomatic FFF patients. In this study, all presented subjects showed overall satisfying functional and radiological outcome.

## Introduction

Flatfoot, or pes planus, is a complex multiplanar deformity characterised by medial rotation and plantar flexion of the talus, eversion of the calcaneus, collapsed medial arch and abduction of the forefoot^1,2^. Flatfoot may be flexible or rigid, depending on whether the appearance of the medial longitudinal arch changes upon weight bearing^1,2^. Flexible flat foot (FFF) is common and physiological in newborns, and related to the fat pad and to the laxity of musculoskeletal structures^1^.

Conservative treatment is preferred in most cases with satisfying results; only in rare, selected, symptomatic, and conservative-refractory cases is surgical treatment proposed^1^. Subtalar arthroereisis (STA) works by elevating the floor of the sinus tarsi to prevent eversion. STA represents the least invasive operative intervention^3^. The goal of STA is to correct FFF by introducing an implant in the sinus tarsi with the objective limiting pronation of the subtalar joint and improving the weight-bearing position of the flat feet. The indications of STA are FFF with hindfoot valgus and medial arch collapse; symptomatic patients that are unresponsive to conservative treatment; and are often used in paediatric patients^4^. Technical simplicity and rapid recovery have been seen as advantageous for this technique and it has evolved into a wide range of implant selection. Other alternative options, such as tarsal fusion or osteotomy, necessitate more extensive surgery, increasing operative risk, and postoperative recovery^5^. Moreover, though a well-established technique, there remains a paucity of information surrounding the effectiveness and outcome.

The aim of this study was to evaluate the radiological and functional outcome of STA in presented cases.

## Materials and Methods

This is a cross-sectional study. We evaluated and followed up eight children with symptomatic FFF, all treated by STA prospectively. The inclusion criteria were defined as children who had (1) symptomatic FFF; (2) were aged 6-13 years; (3) had complete pre- and post-operative clinical and radiological data; and (4) had undergone a minimum of two years of follow-up. Patients with (1) rigid flat feet as a part of neurological disorders or syndromic disease, (2) asymptomatic FFF with only cosmetic problems, (3) without a complete clinical and radiological study, and (4) were treated previously outside our institution were excluded. Informed consent was provided by the patient’s parents or guardian. Patients who met the inclusion criteria were recruited using total sampling method and treated in our institution.

Pre-operative clinical examination includes specific history taking and targeted physical examination focusing on differentiating flexible and rigid flatfoot while simultaneously evaluating any torsional abnormalities on the lower limb, and ligamentous laxity. A 10-point visual analogue scale (VAS scale) was used to assess the level of foot and ankle pain felt by patients in everyday activities.

**Fig. 1:**
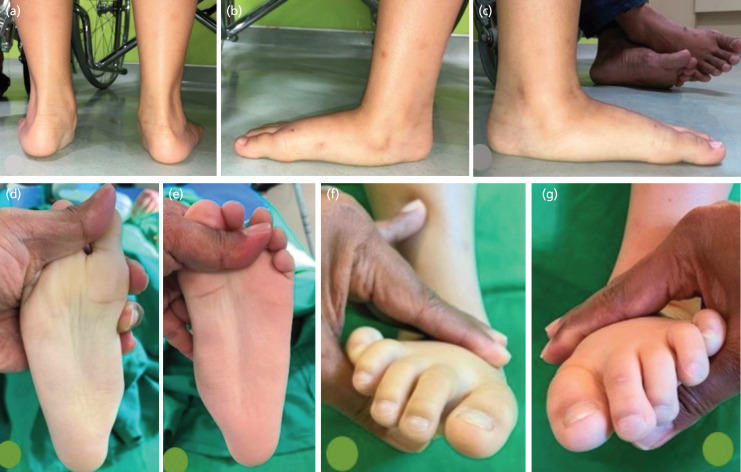
Clinical pre-operative condition to determine the diagnosis of FFF including: hindfoot valgus (A), midfoot sag and loss of medial longitudinal arch on both feet (B, C), convex/bump medial border (D, E), and overpronation (F, G).

Physical findings of the deformed foot were identified (Fig. 1). The toe standing test was performed to assess the ability of the foot to restore the medial arch through the windlass mechanism. Thorough evaluation of the lower limb in terms of rotational deformity of the femur and tibia was conducted. The degree of femoral internal and external rotation (femoral torsion) was measured with the patient placed in the prone position^6,7^. Measuring the torsion of the tibia was performed in a prone position, with the knee flexed to 90°^8^. Heel cord contracture was measured with the Silfverskiöld test to identify the short Achilles tendon that frequently found on symptomatic flatfoot^2,7^. Heel valgus were measured by rearfoot angle using a two-arm goniometer in the double-limb standing position with full weight bearing. The angle was formed by the bisection of the distal one-third of the leg and a longitudinal line that bisected the posterior aspect of the calcaneus. All of the range of motion degrees were measured using a standard goniometer. Laxity was measured based on Beighton scoring.

**Fig. 2:**
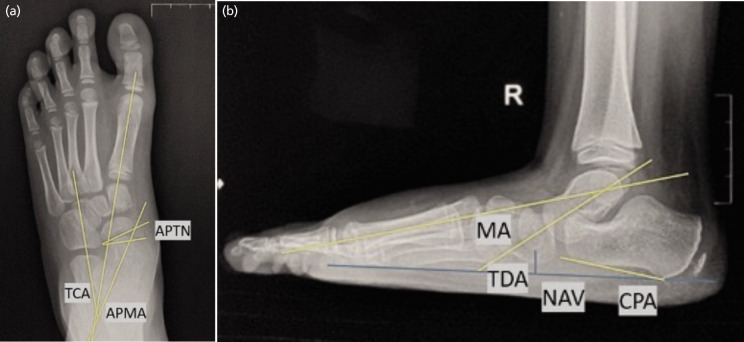
Angle measured in the foot radiograph from AP and lateral view of patient in Fig. 1. Talar bone shifted medially and increased in AP Meary’s angle (11°) and talocalcaneal angle on AP view (31.4°). Overpronation and medial structure of the foot collapsed inferiorly with increased Meary’s angle (21.1°), decreased calcaneal pitch angle (7.4°), and increased in navicular index (12.0) seen on lateral view. The measurement includes AP Meary’s Angle (APMA) normal 3-11 degrees, AP Talonavicular Coverage Angle (APTN) normal <7°, Talocalcaneal Angle (TCA) normal 15°–27°, Lateral Talar-1st Metatarsal or Meary’s Angle (MA) normal 2°– 10°, Calcaneal Pitch Angle (CPA) normal 13°–23°, Talar Declination Angle (TDA) normal 18°–24° and navicular index (length of longitudinal arch divided by navicular height – normally decreased in healthy foot)^9-11^.

For radiographic evaluation, the following angles were measured on both pre- and post-operative weight-bearing foot radiographs to perform a quantitative comparison with the measured angle showed on Fig. 2. We performed full weight-bearing foot radiograph in order to standardise the radiological outcome and level the rotational force of the ankle and foot. The measurement includes AP Meary’s Angle (APMA) normal 3°–11°, AP Talonavicular Coverage Angle (APTN) normal <7°, Talocalcaneal Angle (TCA) normal 15°–27°, Lateral Talar-1st Metatarsal or Meary’s Angle (MA) normal 2°–10°, Calcaneal Pitch Angle (CPA) normal 13°–23°, Talar Declination Angle (TDA) normal 18°–24° and navicular index (length of longitudinal arch divided by navicular height – normally decreased in healthy foot)^9-11^.

**Fig. 3:**
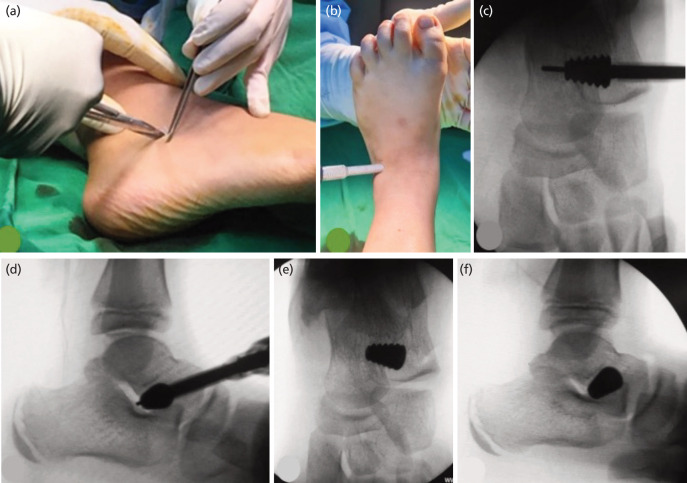
(a) Curved 2-3cm skin incision, direct approach to sinus tarsi. (b) Implant guide insertion. (c and d) Confirmation using image intensifier of the guide wire and implant position on the sinus tarsi. (e and f) Final placement of the implant (at least 1-1.5cm medial to the lateral wall of calcaneus).

Decisions for surgery were all made by a single paediatric orthopaedic surgeon, as well as performing the procedure. All patients were intervened on by general anaesthesia using a lateral approach to the subtalar joint. The procedure was described in Fig. 3 below. We choose the titanium self-locking type Talar-Fit™ [Osteomed, Addison, TX, USA] implant for all patients. We made sure the most medial part of the implant did not cross the bisector line of the talus. ROM of the subtalar joint was examined to be physically normal. A short leg plaster cast was applied with molding the cast to keep heel varus/ valgus alignment, plantigrade foot, and well-supported medial arch. If necessary, additional procedures were performed, including Achilles tendon lengthening (ATL) and gastrocnemius release. Short leg plaster was applied for three weeks or for a longer period of six weeks if additional soft tissue procedures such as ATL were performed. After cast removal, strengthening and range of motion exercises were performed, and the patient could weight bear without aid. During follow-up, no implant removal was necessary if the patient remained asymptomatic from implant-related complications.

Clinical outcomes were measured using a questionnaire collected during pre- and post-operative evaluation. The AOFAS ankle and hindfoot score was used to evaluate preoperative condition and post-operative recovery of ankle and hindfoot function for our FFF patients. It mainly includes three aspects: pain, function, and alignment. The score standard had a maximum of 100 points. A mark of 90–100 was considered as excellent, 75–89 as good, 50–74 as fair, and <50 as poor^9^. Data from the Oxford Ankle Foot Questionnaire for Children (OxAFQ-C) was also administered during a clinical interview before surgery and during follow-up. The questionnaire consists of 15 5-point Likert scale items, 14 of which are used to assess three domains: physical (6 items), school and play (4 items), and emotional (4 items). Response values and their point values include never (4), rarely (3), sometimes (2), very often (1), and always (0). Domain scores were calculated by deriving the sum of each domain and then dividing it by the domain’s maximum value. Better function corresponds to a higher score^12,13^. All of the subjective scoring questionnaire processes were performed by one paediatric orthopaedic surgeon who performed all of the surgery. Post-operative complications were also recorded, including persistent pain, dislocation, and cases of revision.

The statistical analysis describing the radiological and functional outcome differences was done both pre- and postoperatively at the end of follow-up using Student’s t-test for paired samples. All statistical analyses were performed using SPSS v.20.0 for Windows [SPSS, Chicago, IL] with values deemed statistically significant at p<0.05. This study complies with the Declaration of Helsinki, and ethical approval was not required in this case series, as this does not constitute research in our institution. Written informed consent was obtained from the patients' parents/legal guardian for publication and any accompanying images. This study has been reported in compliance with the PROCESS criteria. This study was already registered in the research registry.

## Results

Eight children (15ft) presented in this study. A total of the 8 patients, 5 (62.5%) were male; the rest were female (37.5%), with a mean age at the time of surgery of 10.1±0.83 years. All patients complained of discomfort or soreness in their feet while doing high-intensity activities such as running and jumping. Unbalanced gait was also reported and clearly seen while patients were getting fell easily, especially in uneven terrain, and had difficulty in sports participation. The mean follow-up of the feet was 42.6±9.69 months. Three patients underwent adjunct procedures (3 ATL) due to equinus that accompanied the planovalgus deformity. The clinical data of the patients were showed in Table I.

**Table I:** Demographic summary of clinical evaluation and procedure to all subjects in presented case.

No.	Sex	Age	Main Complaint	Ankle Dorslflexlon (o)		Tibial Ext. Rotation (o)		Femoral Int. Rotation (o)		Belghton Score	Other Diagnosis/ Condition	Adjunct Procedure
				L	R	L	R	L	R			
1	M	13	Pain, unstable feet while walking	6	5	35	35	37	35	2	Short/Tight Achilles tendon	Bilateral ATL (White-Hoke)
2	M	10	Pain	20	18	30	30	45	45	0	WA	N/A
3	M	11	Pain, fell down easily	3	7	30	30	42	42	2	Achilles tightness	Bilateral ATL (White-Hoke)
4	F	13	Pain, unstable feet while walking	6	6	25	25	45	45	2	Achilles tightness	Bilateral ATL (White-Hoke)
5	F	6	Discomfort, during prolong walking/ standing	15	15	27	27	65	70	7	Increasing femoral anteversion generalized laxity	N/A
6	M	10	Pain, gait disturbance	17	16	25	25	40	42	0	N/A	N/A
7	F	10	Pain, gait disturbance	15	15	25	25	40	40	8	Generalized laxity	N/A
8	M	8	Pain	15	-	30	-	40	-	0	N/A	N/A

Notes - M: male, F: female, N/A: not available

Post-operative results showed significant increases in both AOFAS and OxAFQ-C subjective scoring with a p-value of <0.001 for both (Table II). AOFAS score showed a “good” level of satisfaction after surgery (mean 87.0±2.29) compared to “fair” result before surgery (mean 59.1±1.83). OxAFQ-C score improved in all domains, including physical, school and play, and emotional (mean 21.7±0.81 vs 42.6±2.59). Although pain was mild to moderate pre-operatively, it was significantly lowered after surgery. Objective correction of the deformity, clinically, can also be identified by evaluating the decreasing of the valgus heel angle after STA during follow-up. Degree of correction was statistically significant from pre- and post-operative comparisons (p=0.001).

**Table II:** Comparison of clinical outcome pre- and post-operative among subjects. Patient’s complaint regarding remaining symptom or complication that may occurred also being evaluated.

No	Sex	Age	Follow-up	AOFAS (score)		OxAFQ-C (score)		Valgus heel - Right foot (o)			Valgus heel - Left foot (o)			Complication
		(years)	(mon)	Pre-	Post-	Pre-	Post-	Pre-	Post-	Change	Pre-	Post-	Change	
1	M	13	58	55	84	22	41	12.5	5	7.5	14	7	7	N/A
2	M	10	53	65	91	21	40	3	2	1	2	2	0	N/A
3	M	11	46	52	84	20	41	12	8	4	15	7.5	7.5	N/A
4	F	13	46	58	85	19	42	6	6.5	-0.5	7	3.5	3.5	N/A
5	F	6	36	60	77	20	29	17.5	7	10.5	25	12	13	Implant
														migration
6	M	10	36	65	94	22	44	11.5	3.5	8	18	5.5	12.5	N/A
7	F	10	36	54	84	24	52	8	2	6	9	5	4	N/A
8	M	8	30	64	97	26	52	*	-	-	16	7	8	N/A
		Mean		59.1ab1.83	87.Ot2.29	21.7afc0.81	42.6±2.59							
		P value		<0.001		<0.001					0.001			

Notes - *p value was obtained with paired T-test. N/A: Not applicable

**Table III:** Radiographic parameter comparison between pre-operative and last follow-up values in feet treated with subtalar arthroereisis.

	Sex	Left/Right	MA (°)		CPA (°)		TDA (°)		NAV (proportion)		APMA (°)		APTN (°)		TCA (°)	
			Pre-	Post-	Pre-	Post-	Pre-	Post-	Pre-	Post-	Pre-	Post-	Pre-	Post-	Pre-	Post-
1	M	Right	11.5	8.4	4	15	17	9.5	8.3	4	5	6.3	9	7.7	28.5	22.3
		Left	23	10	0.5	9.5	26	13	10	4.9	7.2	5.5	5.4	5	30	25
2	M	Right	8.2	7	10.8	15	29.2	22.4	8.1	4.2	9.5	10	30	15	25.1	22
		Left	12.1	9.4	10.4	17	32.6	18	10.5	5.1	22.3	15	33	17	37.2	30
3	M	Right	12.1	7	8	13.4	25	22	12.5	5.4	4.2	2.3	10	4	26.5	23
		Left	17.5	8.5	7.2	9	32	20.4	20	6	7.8	3	2	2	27.5	25.4
4	F	Right	39.5	9.2	-18.5	4.5	55	25	45	12	10.2	20	25	-27	24.2	21.5
		Left	30.3	4.8	-7.5	5	45	19.2	44	8.8	0.3	30	29	-18	22	11.5
5	F	Right	21.1	23.5	7.4	7.5	47	40.2	12	9	11	15	41	56.5	31.4	36
		Left	10.2	19.2	13.5	4	32	39.8	7.6	7.5	9.3	21.5	39	55.5	25.2	33.3
6	M	Right	10.5	2	13.5	16.2	39.5	19.3	13	5.2	5	2	5.1	3	22.4	15
		Left	32	6.2	10	16	42.5	23.5	12.5	6	14	1.5	19.3	4.5	22	18.3
7	M	Left	19	8	9	14	29	18	30	9.2	24	4	12	4	12	16
8	F	Right	22	6	7	15	24	12	12	5.2	11	10	10	2	19	14
		Left	24	4	6	16	29	16	14	6.4	10	6	6	3	22	16
	Mean		19.5±2.37	8.8±1.43	5.4±2.18	11.8db1.21	33.6±2.62	21.2±2.26	17.3±3.19	6.5±0.58	11.6±2.08	10.4±2.17	18.3±3.43	8.9±5.96	25.0±1.50	21.9±1.84
	% Cl		4.4-16.86		-10.2-(-2.49)		7.33-17.52		4.80-16.61		-5.65-8.01		-0.99-19.87		0.26-0.58	
	P value		0.002		0.003		<0.001		0.002		0.717		0.073		0.034	

Notes - *p value was obtained with paired T-test, MA: Meary’s angle, CPA: calcaneal pitch angle, TDA: talar declination angle, NAV: navicular index, APMA: anteroposterior Meary’s angle, APTN: AP talonavicular coverage angle, TCA: talocalcaneal angle

**Fig. 4:**
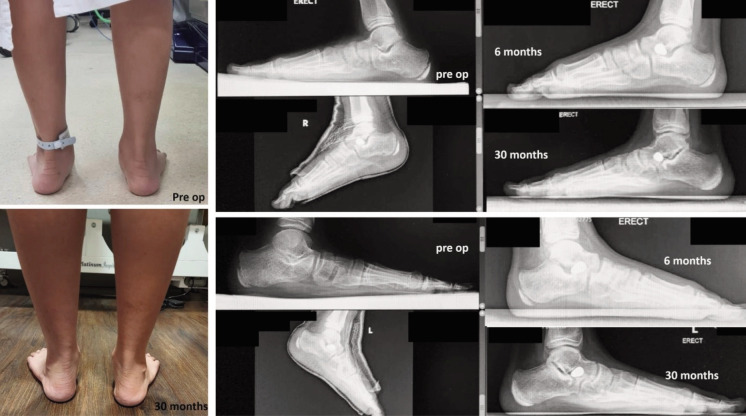
Clinical and radiographic conditions of one patient in follow-up of 6 and 30 months.

The longest patient that we evaluated in this study (58 months follow-up time) showed improvement in AOFAS and OxAFC scores with a 7.5° and 7.0° clinical heel valgus angle correction. Subjectively, the patient felt an increased ability to do daily activities without pain on both of his feet. No infection, implant loosening, or persistent pain was reported in this patient. Clinical and radiographic results could be seen in Fig. 4.

**Fig. 5:**
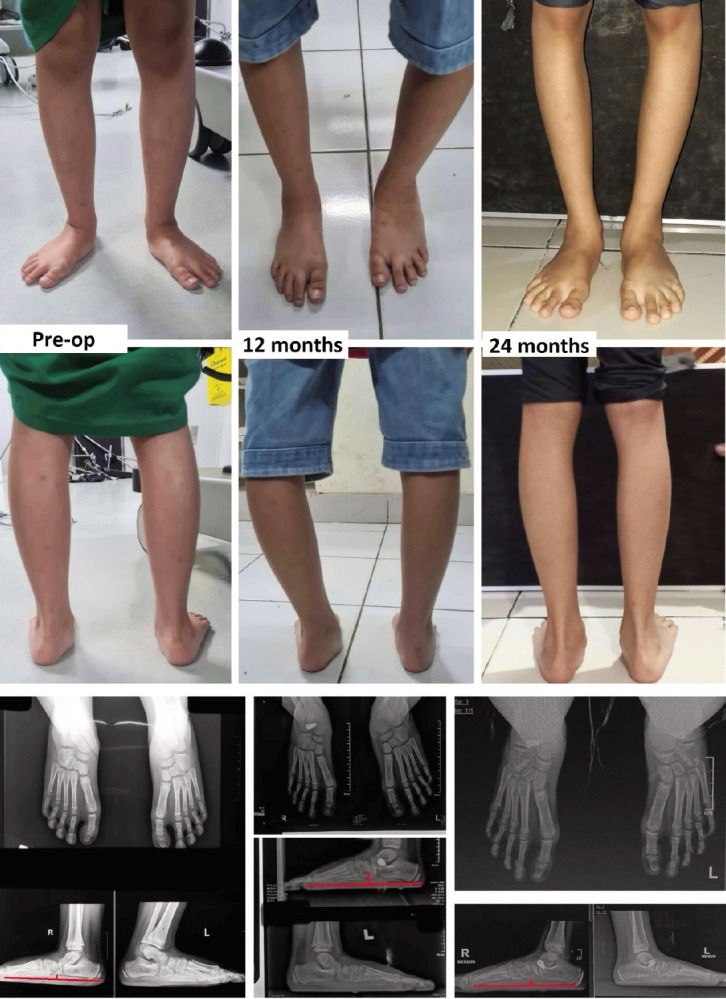
Pre-operative clinical condition, 12 months, and 24 months follow-up of FFF patient with increased femoral anteversion (both the patella was internally rotated). Radiographic evaluation at 24 months of follow-up showed implant migration to the lateral of the right foot (loosening seen on lateral radiograph). The navicular index was increased indicating an overpronated foot and loss of longitudinal arch.

One patient had their valgus angle increased by 0.5° instead of being corrected (patient number 4 in Tables II and III). However, his clinical questionnaires were improved. This patient had a negative pre-operative CPA angle related to posterior tightness that prevented ankle dorsiflexion that caused hindfoot overloading. One patient with bilateral FFF and increased femoral anteversion had implant migration on her right foot (patient number 5). The implant migrated to the lateral on two years follow-up, and the APTN returned to pre-operative condition. The navicular index increased, indicating the navicular height collapsed and the foot became pronated again. However, during follow-up the patient refused revision surgery for this complication due to already satisfied with the result (Fig. 5).

From the radiological evaluation, we observed correction in all angles measured (Table II). A comparison of radiographic outcomes from the lateral view showed that the lateral talar-first metatarsal (Meary’s) has a correction degree from a mean of 19.5° ± 2.37° to 8.8° ± 1.43°, CPA from 5.4° ± 2.18° to 11.8° ± 1.21°, TDA from 33.6° ± 2.62° to 21.2° ± 2.26°, and the navicular index proportion decreased from 17.3° ± 3.19° to 6.5° ± 0.58°. There were significant differences between pre- and post-operative lateral radiographic measurements in this study with p-values of 0.002, p=0.003, p<0.001, and p=0.002 of MA, CPA, TDA, and NAV, respectively. A comparison of radiographic outcomes from the anteroposterior view showed that the AP talar-first metatarsal (Meary’s) angle has a correction degree from a mean of 11.6° ± 2.08° to 10.4° ± 2.17°, talonavicular coverage from 18.3° ± 3.43° to 8.9° ± 5.96°, and TCA from 25.0° ± 1.50° to 21.9° ± 1.84°. Although only TCA was statistically significant (p=0.034). During the follow-up study, no implant was yet to be removed because the patients remained asymptomatic from implant-related complications.

## Discussion

STA is one of the surgical techniques in cases of symptomatic FFF. Previously, calcaneal lengthening with medial displacement calcaneal osteotomy (MDCO) and lateral column lengthening (LCL) was proposed as the first option in symptomatic FFF^2,5^. STA has several advantages, which include being minimally invasive, providing a three-dimensional correction, and its rapid recovery, while soft tissue procedures lack satisfactory outcomes, and osteotomies often do not address the deformity correction intended with the risk of non- or malunion and a longer time to recover^1,9,14^. Despite controversial results and poor evidence of previous studies, STA in our reported cases proved its reliability in treating FFF with minimal complication, fast recovery, and notable correction both in clinical and radiological parameters.

A study by Fernandez *et al* showed STA is an option besides osteotomy for valgus hindfoot in FFF patients^15^. Chong *et al* compared LCL with STA in their prospective study with one year of follow-up resulted in a similar outcome but faster recovery. Higher levels of evidence, such as randomised control trials (RCTs), also suggested to conclude the superiority^5^. In our study, all subjects had significant improvement in lateral radiographic measurements (p<0.05). A study by Graham *et al* showed an 80% satisfaction score using the Maryland foot score with a 5.7° TDA correction and a 0.8° CPA correction after 5 years, while De Pellegrin, in his study, revealed 93.7% satisfaction 4.5 years after surgery with 3° and 18° TDA and CPA corrections, respectively^16,17^.

Our study used two different questionnaires to evaluate clinical outcomes. AOFAS questionnaire used in the study evaluating the outcome of STA by Indino *et al* resulted in a mean post-operative score of 97.3 from 56 patients included in their study. Oxford questionnaire for children is also being utilised in other outcome-based studies, such as the one reported by Ruiz-Picazo *et al* in their report from 32ft, which showed a 7.7 mean improvement in the OxAFQ score. A total of 15ft were included in our study, and follow-up results showed a significant increase in both AOFAS and OxAFQ-C subjective scoring (p<0.001). Despite not all patients having their feet fully corrected based on heel valgus and radiological angles, but overall, eight children showed a high degree of satisfaction after STA. Our findings are consistent with the aforementioned literature on STA outcomes^4,18^.

Timing of the surgery is important because physiological deformity can be seen in children aged 3-5 years old, where the arch develops naturally by the middle of childhood (approximately age 5 years)^19^. Over the increased age, the incidence of flatfoot was found to be decreased from 70% at ages 3-4 years to 9.1% at age of 720. This prevalence data showed us the progression of the disease, which is part of the normal development of a child’s foot; therefore, clinical judgment for surgery is crucial. Ruiz-Picazo *et al*, on their study stated that surgery is recommended to be performed between the ages of 8 and 12 years: all our patients were intervened at ages of 6 to 13 years^18^. Spontaneous correction was still expected in younger ages, while surgery performed on those over 12 years old tends to be exceptional, since the aim of arthroereisis is to correct the position of the talus related to the calcaneus hence enabling the remodelling of these bones and the subtalar joint following children’s growth. Two years are required for the purpose; this means that, as the patient’s age gets older, less time remains for the bones and ligaments of the hindfoot to be remodeled^21^.

STA is often accompanied by adjunct soft tissue procedures to achieve full correction and improve outcomes. Lengthening of the tight heel cord is one of the procedures that performed by most surgeons since 27% flatfoot cases have short Achilles tendon that require surgical treatment. All of the subjects in this study have their ROM angle measured and documented to determine the need for tendon lengthening as an adjunct procedure of STA. Despite quite a routine procedure to be performed, evidence regarding its substantial effect is still lacking. Some studies otherwise have positive outcome. Lin *et al* showed that ATL can correct the cavus deformity and improve the calcaneus eversion and forefoot supination combined with STA, similar to the study by Cicchinelli *et al*, using a gastrocnemius recession as an adjunct procedure, which displayed a positive effect on the correction of transverse plane deformity combined with STA^22,23^. Three patients in our study underwent Achilles tendon lengthening while others did not. Pre-operative evaluation is essential prior to correction of paediatric flatfoot because untreated equinus heel can promote failure of flatfoot correction^3^.

Two patients in our study showed an increase in generalised laxity; one foot had the medial bump not fully corrected (negative values of CPA and TDA). Despite this result, the clinical outcome was still improved and did not affect daily activities. FFF can occur due to or result from generalised laxity (musculoligamentous theory). Lin *et al*, stated that generalised laxity could be found in moderate to severe cases of FFF. However, the direct correlation of ligamentous laxity and degree of correction is still unknown^22^.

Lower limb rotational anomaly is very common (femoral anteversion, tibial external rotation). Cebulski-Delebarre *et al* stated that secondary external tibial torsion is a partial compensation for patients with femoral anteversion^24^. According to Zafiropoulos *et al* there is a positive association between excessive hip internal rotation and flat feet^25^. In this study, one patient with the lowest radiological angle of correction had a loosened implant in a two-year follow-up, possibly due to malrotation of the whole lower limb. She had an increase in pre-operative femoral anteversion, resulting in increased hindfoot pressure in valgus heel, talus adducted, forefoot abducted and pronated, so in the long term, she had her implant migrate laterally. Still, there is no clear evidence for this direct effect of torsional deformity of the lower limb on implant loosening and whether there is a need for surgical correction^25^.

STA using many types of implants have been done in recent years but still there is lack of data to determine the optimal choice for treatment due to a shortage of high quality, long-term follow-up studies. Studies addressing metallic implants are limited and more data are needed to understand it’s effect on paediatric foot. A study by Moraca *et al* in 74 paediatric FFF showed that a metallic device in STA provided low complication rates and high patient satisfaction in their 10 years follow-up study^26^.

Despite the results in this study, there were limitations such as a small number of patients, absence of a control group, single-centre nature, and also the study did not compare STA with other surgical techniques or interventions, making it challenging to assess the relative merits and drawbacks of STA in relation to alternative treatment options. A longer-term follow-up at least until skeletal maturity would provide more comprehensive insights of STA since one patient developed implant loosening. Further investigation and research with comparative studies could be done in the future, assessing the durability and effectiveness of STA.

## Conclusion

STA is an option for surgical treatment in symptomatic FFF patients. In this study, all presented subjects showed overall satisfying functional and radiological outcome. Future investigation and research with comparative studies could be done to assess the durability and effectiveness of STA.
